# Clinical Diagnostic and Prognostic Potential of *NDRG1* and *NDRG2* in Hepatocellular Carcinoma Patients

**DOI:** 10.3389/fonc.2022.862216

**Published:** 2022-06-20

**Authors:** Shaohua Xu, Ruihuan Gao, Yidan Zhou, Ying Yang, Yi Zhang, Qianyuan Li, Chunhua Luo, Song-Mei Liu

**Affiliations:** ^1^ Department of Clinical Laboratory, Center for Gene Diagnosis & Program of Clinical Laboratory, Zhongnan Hospital of Wuhan University, Wuhan, China; ^2^ The First College of Clinical Medical Science, Three Gorges University, Hubei, China

**Keywords:** LIHC, prognosis, *NDRG1*, *NDRG2*, hepatocellular carcinoma, methylation, Kaplan–Meier

## Abstract

**Background:**

Primary liver cancer is still the most common lethal malignancy. The N-myc downstream-regulated gene family (*NDRG1–4*) is a group of multifunctional proteins associated with carcinogenesis. However, systematic evaluation of the diagnostic and prognostic values of *NDRG1* or *NDRG2* expression in liver cancer is poorly investigated.

**Method:**

The gene expression matrix of liver hepatocellular carcinoma (LIHC) was comprehensively analyzed by the “limma” and “Dseq2” R packages. The Gene Ontology (GO) and Gene Set Enrichment Analysis (GSEA) were used to identify the biological functional differences. A single-sample GSEA (ssGSEA) was conducted to quantify the extent of immune cell infiltration. Finally, the clinical and prognostic information of LIHC patients was systematically investigated using Kaplan–Meier analysis and logistic and Cox regression analysis.

**Results:**

Compared with normal tissues, *NDRG1* expression was higher, whereas *NDRG2* expression was lower in tumor tissues (*P <*0.001). The area under the receiver operator characteristic curve (AUROC) of *NDRG1* and *NDRG2* for LIHC was 0.715 and 0.799, respectively. Kaplan–Meier analysis revealed that *NDRG1* and *NDRG2* were independent clinical prognostic biomarkers for the overall survival (OS, *P* = 0.001 and 2.9e−06), progression-free interval (PFI, *P* = 0.028 and 0.005) and disease-specific survival (DSS, *P* = 0.027 and *P <*0.001). The C-indexes and calibration plots of the nomogram suggest that *NDRG1* and *NDRG2* have an effective predictive performance for OS (C-index: 0.676), DSS (C-index: 0.741) and PFI (C-index: 0.630) of liver cancer patients. The mutation rate of *NDRG1* in liver cancer reached up to 14%, and DNA methylation levels of *NDRG1* and *NDRG2* promoters correlated significantly with clinical prognosis.

**Conclusions:**

The mRNA expression and DNA methylation of *NDRG* superfamily members have the potential for LIHC diagnosis and prognosis *via* integrative analysis from multiple cohorts.

## Introduction

Primary liver cancer is the fourth leading cause of cancer-related deaths worldwide, characterized by an insidious onset and a low rate of early diagnosis (1). In China, liver hepatocellular carcinoma (LIHC) is the most common type of primary liver cancer and typically results from chronic hepatitis B virus infection (2). Despite the recent advances in diagnostic and therapy, the 5-year relative survival rate of LIHC remains to be only 12% (3). Currently, LIHC contributes to over half of the new deaths, and it is predicted to rise continuously in the next decade (4). Therefore, identifying potential biomarkers that improve diagnostic accuracy and prognostic prediction is critical.

Increasing evidence indicates that disturbance of proto-oncogenes and tumor suppressor genes results in hepatocarcinogenesis, which is a complicated pathophysiological process (5). Viral infection and metabolic stress induce genetic and epigenetic alterations through cell cycle turnover and the inflammatory environment (6). The N-myc downstream-regulated gene (*NDRG1–4*) family, a hypoxia-associated protein, has been involved in cell proliferation and differentiation, stress responses, tumor progression, and metastasis (7–9). *NDRG1* is involved in cellular skeleton modification and organ development and can be induced by hypoxia and DNA damage (7, 10). *NDRG2* is highly expressed in dendritic cells and maintains activated leukocyte adhesion (11). Moreover, *NDRG3* could promote cell growth and angiogenesis and participate in the lactate-dependent hypoxia signaling pathway (12). *NDRG4* is exclusively expressed in the embryonic stage and regulates the proliferation and growth of nerve cells and cardiomyocytes (13). Of note, abnormal expression of *NDRG1* or *NDRG2* has been found in different cancer types, such as in esophageal cancer, colorectal cancer, breast cancer, prostate cancer, and hepatocellular carcinoma, and is significantly associated with poor prognosis (10, 14–16). Existing data indicate that *NDRG1* is upregulated and *NDRG2* is downregulated in LIHC. However, a few studies focused on the diagnostic and prognostic values of *NDRG1* or *NDRG2* in LIHC, and their potential mechanisms in LIHC remain unknown.

Here, the diagnostic and prognostic significance of *NDRG1* and *NDRG2* were systematically identified in LIHC using RNA-seq data from the TCGA database. We first comprehensively analyzed the gene expression matrix of LIHC and then applied bioinformatics methods (namely, Gene Ontology (GO) terms, Kyoto Encyclopedia of Genes and Genomes (KEGG) pathway analysis, and Gene Set Enrichment Analysis (GSEA)) to explore the underlying biological mechanism. Secondly, we analyzed DNA mutation and methylation in *NDRG1* or *NDRG2*, and explored the relevance between immune cells and *NDRG1* or *NDRG2* expression by the single sample GSEA (ssGSEA). Moreover, clinical and follow-up information of LIHC patients were used for Kaplan–Meier analysis, and logistic and Cox regression analysis. Finally, we plotted a nomogram for the prognosis prediction of the patients. Taken together, this study revealed that *NDRG1* or *NDRG2* could be a potential diagnostic and prognostic biomarker for LIHC.

## Materials and Methods

### Data Source

RNA-seq data for pan-cancer analysis were retrieved from the UCSC XENA (https://xenabrowser.net/datapages/) (17, 18). The mRNA expression datasets (GSE14520, GSE25097, and GSE36376) were obtained from the GEO database (https://www.ncbi.nlm.nih.gov/geo/) to further validate the results of pan-cancer analysis in LIHC patients. Meanwhile, we also collected the data for NDRG1 and NDRG2 protein expression from the Human Protein Atlas (HPA) (https://www.proteinatlas.org/). The mRNA expression matrix file of TCGA-LIHC and the corresponding clinical data were collected from the TCGA website (https://portal.gdc.cancer.gov/repository), and level-3 HTSeq-FPKM data were transformed into TPM (transcripts per million reads) for subsequent analyses. A total of 371 patient information was used, while unavailable or unknown clinical information was excluded.

### Clinical Specimen Collection, RNA isolation, and qPCR

Thirty-two pairs of fresh-frozen tissues (LIHC tissues and their adjacent tissues) were collected from the Zhongnan Hospital of Wuhan University with informed consent and approval from the hospital ethics committee. cDNA was synthesized from total RNA using the PrimeScript RT Reagent Kit (Vazyme, R333-01, China). The SYBR Prime Script RT-PCR kit (Vazyme, Q712-02, China) was used for qPCR on a CFX96 Connect Real-Time System (Bio-Rad, America). The primer sequences were as follows: *NDRG1*-F, 5′-GAAGTGGTCCACACCTACCG-3′; *NDRG1*-R, 5′-GTCCGCCATCTTGAGGAGAG-3′; *NDRG2*-F, 5′-GCCCAGCGATCCTTACCTAC-3′; *NDRG2*-R, 5′-TGCAAGCTGGTCCAGAGATG-3′; *GAPDH*-F, 5′-GGAGCGAGATCCCTCCAAAAT-3′; and *GAPDH*-R, 5′-GGCTGTTGTCATACTTCTCATGG-3′.

### Identification of the *NDRG1* and *NDRG2* Expression Profile

The raw expression profiles from GEO datasets were preprocessed by R software (version 4.0.5) and differentially analyzed by running the “limma” R package (version 3.46.0). Gene expression data of LIHC case samples were stratified into high- and low-expression groups based on the median expression of *NDRG1* and *NDRG2*, respectively. The expression analysis between high- and low-expression groups was performed using the “DESeq2” R package (19) (version 3.18.1; http://www.Rproject.org). Genes with the threshold for | log2FoldChange | >0.5 and adjusted *P <*0.05 were regarded as statistically significant.

### Functional Annotation and Enrichment Analysis

R package ClusterProfiler (version 3.14.3) was applied to GO term analysis, KEGG pathway analysis, and GSEA to elucidate the function and pathway differences between the high- and low-expression groups (20, 21). Here, the curated gene sets (c2.cp.v7.2.symbols.gmt) from MSigDB Collections were selected as reference gene sets for GSEA. A permutation test with 1,000 iterations was used to identify pathways that had changed significantly. Significant pathway enrichment was identified by a false discovery rate (FDR) <0.25 and *P*-value <0.05.

### Single-Sample GSEA (ssGSEA) for Immune Infiltration Analysis

The ssGSEA was conducted using the GSVA R package (3.6.3) to quantify the tumor immune infiltration levels in 24 types of immune cells (22) and the marker of which was obtained from a previous study (23). Next, Spearman’s correlation analysis was conducted to evaluate the associations of *NDRG1* and *NDRG2* expression with immune cell infiltration, and the Wilcoxon rank-sum test was used to investigate the enrichment scores of immune infiltration levels between high- and low-*NDRG1* and *NDRG2* expression groups.

### DNA Mutations and Methylation Analysis

The cBio Cancer Genomics Portal (http://cbioportal.org) was applied to analyze *NDRG* family alterations in the TCGA-LIHC sample, which is an interactive exploration website of multidimensional cancer genomic datasets (24). The DNA CpG methylation of *NDRG1* and *NDRG2* in TCGA was also analyzed by MethSurv (https://biit.cs.ut.ee/methsurv/) to explore the relevance between the CpG methylation level and prognostic values (25).

### Statistical Analysis

The R software (version 4.0.5) was used for all statistical analysis and graphical plotting. Scatter plot analysis was performed using the Wilcoxon rank-sum test. Non-parametric survival analysis was performed by the Kaplan–Meier method and log-rank test. Correlation analysis was evaluated using the Spearman’s coefficient. The Wilcoxon signed rank test, Kruskal–Wallis test, and Chi-Squared test were used to assess the clinicopathological features between high- and low-expression groups. The diagnostic performance of *NDRG1* or *NDRG2* expression was tested by the Area Under the Receiver Operator Characteristic Curve (AUROC). Univariate and multivariate analyses using Cox proportional hazard modeling were performed to estimate the death risk. Unless stated otherwise, *P <*0.05 (two-sided) was considered statistically significant.

## Results

### *NDRG1* Was Upregulated and *NDRG2* Was Downregulated in LIHC

**Figure 1:**
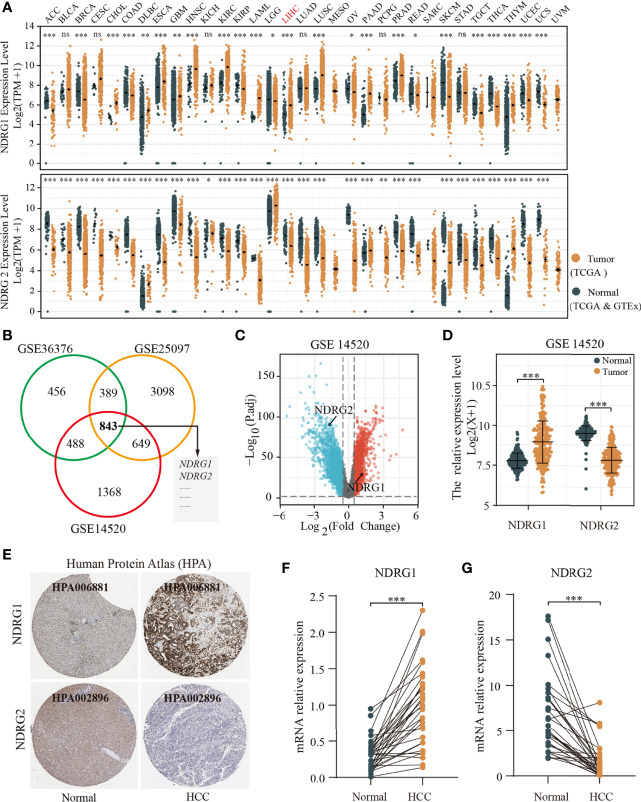
Differential mRNA expression profiles of *NDRG1 or NDRG2* in different cancer types. **(A)** The expression levels of *NDRG1* and *NDRG2* in different cancer types were analyzed based on the TCGA database. **(B)** Venn plots of DEGs (| log2FoldChange>0.5 and adjusted *P <*0.05) identified in three GEO databases. **(C)** The volcano plots for all the coding genes obtained in the GSE14520 database. Red and blue dots represent up-regulated DEGs and down-regulated DEGs, respectively. **(D)** The scatter diagram of *NDRG1* and *NDRG2* mRNA expression levels in GSE14520 database. **(E)** The protein expression of *NDRG1* and *NDRG2* in LIHC was obtained from the Human Protein Atlas (HPA, https://www.proteinatlas.org/). **(F, G)** The expression levels of *NDRG1* and *NDRG2* were obtained by qPCR assay in liver carcinoma and para-carcinoma tissues. *P < 0.05; **P < 0.01; ***P < 0.001. ns, no significance.

We used pan-cancer RNA-seq data from the UCSC XENA to evaluate the mRNA expression levels of *NDRG1* and *NDRG2* in diverse human cancers. As shown in **Figure 1A**, the mRNA expression of *NDRG1* and *NDRG2* was found to be significantly different (*P <*0.001) in almost all tumor types, including LIHC. To further verify the above results, we performed common differential expression gene analysis among the three selected GEO datasets (GSE14520, GSE25097, and GSE3637), and *NDRG1* and *NDRG2* were found to be two of the 843 overlapping genes (|log2FoldChange| >0.5, P*adj* <0.05) (**Figure 1B**). As shown in **Figures 1C, D**, the results from GSE14520 showed that *NDRG1* mRNA was highly expressed in LIHC tissues compared to the adjacent normal tissues (*p <*0.001), and *NDRG2* mRNA was markedly downregulated in LIHC tissues (*P <*0.001).

To further validate NDRG1 and NDRG2 protein expression in LIHC, we analyzed the NDRG1 and NDRG2 expression profiles in the HPA database (**Figure 1E**). The immunohistochemical staining from HPA also showed that NDRG1 and NDRG2 proteins were expressed in a pattern consistent with the mRNA-level changes in LIHC tissues. At the experimental level, we analyzed *NDRG1* and *NDRG2* mRNA expression by qPCR in 32 pairs of LIHC tissues (**Figures 1F, G**). The qPCR results again indicated the high expression of *NDRG1* (*P <*0.001) and low expression of *NDRG2* (*P <*0.001) in LIHC tissues.

### DNA Mutation and Methylation of *NDRG1* or *NDRG2* for LIHC Prognosis

**Figure 2:**
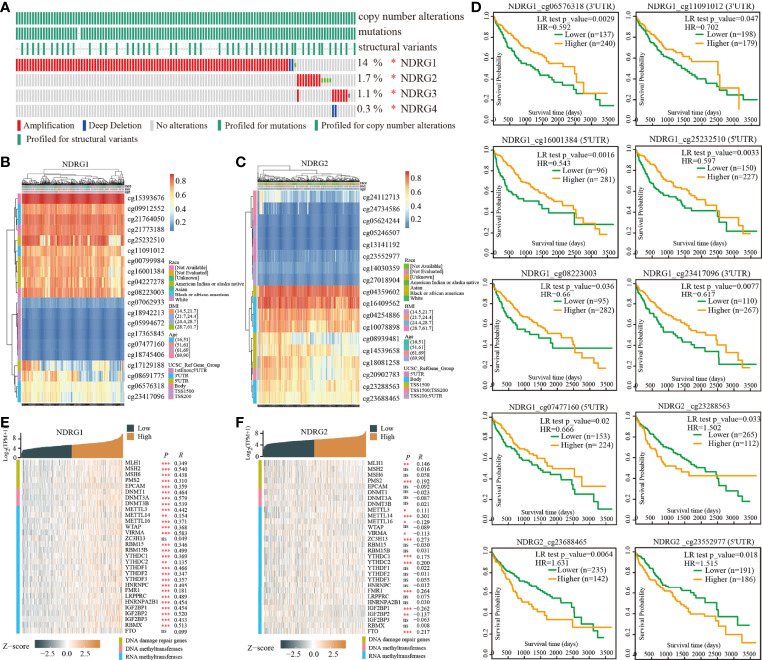
The analysis of DNA mutation and methylation of *NDRG1 or NDRG2*. **(A)** The overview of DNA mutation of the *NDRGs* family genes, mapped by cBioPortal (http://www.cbioportal.org/). **(B, C)** The DNA methylation clustered expression of *NDRG1* and *NDRG2* by using MethSurv^©^2017 (https://biit.cs.ut.ee/methsurv/). Red to blue represents a high to low expression. **(D)** The significantly prognostic values of the promoter CpGs in *NDRG1* and *NDRG2* obtained from MethSurv^©^2017. **(E, F)** The heatmaps of *NDRG1* and *NDRG2* expression in relation to DNA repair genes and methyltransferases. *P < 0.05, **P < 0.01, ***P < 0.001. NS, no significance.

Next, we analyzed the mutation frequencies of *NDRG1* or *NDRG2* in TCGA-LIHC using the cBioPortal online tool. As shown in **Figure 2A**, a high mutation rate of *NDRG1* (up to 14%) was observed in LIHC patients, while other *NDRG* family genes (*NDRG2*, *NDRG3*, and *NDRG4*) were rarely mutated (less than 2%). Moreover, we analyzed the DNA methylation levels of *NDRG1* and *NDRG2* (**Figures 2B, C**) and the prognostic values (**Figure 2D**) of single CpG in TCGA-LIHC *via* MethSurv^©^2017. As a result, they showed that cg15393676 in *NDRG1* (**Figure 2B**), and cg16409562 and cg04359602 in *NDRG2* (**Figure 2C**) showed the highest methylation in their promoter regions. The DNA methylation level has been negatively correlated with the gene expression level. As shown in **Figure 2D**, LIHC patients with hypermethylation in *NDRG1* and hypomethylation in *NDRG2* could have a better clinical prognosis, which supported our mRNA expression results.

Furthermore, we explored the relationship between the expression of *NDRG1* or *NDRG2* and DNA repair and methyltransferase genes. As shown in **Figure 2E**, *NDRG1* expression has a strong positive correlation with DNA repair genes and methyltransferase genes in LIHC. As shown in **Figure 2F**, we found that *NDRG2* expression has a markable positive correlation with only a few RNA methyltransferase genes and DNA repair genes, but not with DNA methyltransferase in LIHC. This suggests that *NDRG1* may indirectly affect the development and progression of LIHC by regulating epigenetic status.

### *NDRG1-* or *NDRG2*-Related Differentially Expressed Genes

**Figure 3:**
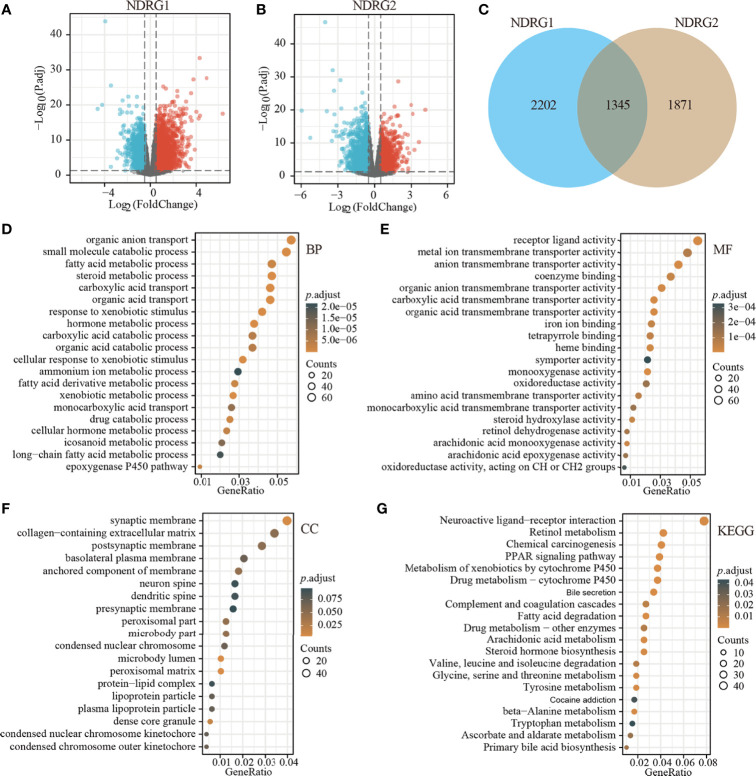
Identification of DEGs in LIHC tissues with low- and high-expressed *NDRG1* and *NDRG2* groups. **(A, B)** Volcano plot of DEGs between low- and high-*NDRG1* and *NDRG2* expression groups based on the TCGA & GTEx datasets. **(C)** Venn diagram of the identified DEGs between low- and high-*NDRG1* and *NDRG2* expression groups. **(D–G)** The plots for the results of enriched GO terms and KEGG pathway analysis using the “ClusterProfiler” R package. The x-axis represents the proportion of DEGs, and different circle sizes represent the number of DEGs.

The enrolled 371 LIHC tumor samples from the TCGA dataset were stratified into high- and low-expression groups according to *NDRG1 or NDRG2* median values (cut-off value of 50%), respectively. The identified differentially expressed genes (DEGs) (|log2FoldChange| >0.5, *Padj <*0.05) between different cohorts were illustrated by the volcano plot. A total of 3,547 genes (2,322 upregulated and 1,225 downregulated) were identified as DEGs in the high-*NDRG1* group (**Figure 3A**), and 3,216 genes (1,204 upregulated and 2,012 downregulated) in the high-*NDRG2* group (**Figure 3B**). As shown in **Figure 3C**, 1,345 overlapping DEGs were found between the *NDRG1* and *NDRG2* groups based on the TCGA-LIHC dataset.

To predict the function of *NDRG1*- and *NDRG2*-related DEGs, we conducted GO and KEGG pathway analysis using 1,345 overlapping DEGs by the “ClusterProfiler” R package (version 3.14.3). As shown in **Figures 3D–G**, we found that *NDRG1*- and *NDRG2*-related DEGs were involved in many biological processes, namely, retinol metabolism, bile secretion, fatty acid degradation, chemical carcinogenesis, amino acid metabolism, PPAR signaling pathway, and the cytochrome p450 pathway.

### *NDRG1-* or *NDRG2*-Related Signaling Pathways

**Figure 4:**
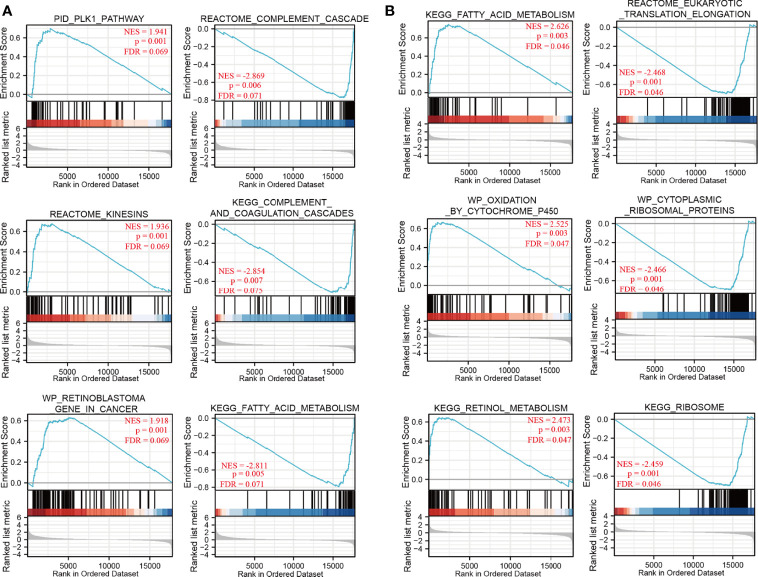
The gene set enrichment analysis of *NDRG1* and *NDRG2.* **(A, B)** Representative enrichment plots from GSEA. Several pathways and biological processes were differentially enriched in *NDRG1 and NDRG2*-related hepatocellular carcinoma. NES, normalized enrichment score; p.adj, adjusted P-value; FDR, false discovery rate.

To identify *NDRG1*- and *NDRG2*-related signaling pathways, the GSEA was then conducted between low- and high-expression groups, based on significant differences (*P*-value <0.05, FDR <0.25) in the enrichment of the MSigDB Collection [c2.cp.v7.2.symbols.gmt (curated)]. Here, the most significant enrichment was selected according to the Normalized Enrichment Score (NES). As shown in **Figure 4**, GSEA results showed that the *NDRG1*-related signaling pathway is involved in the PLK1 pathway, complement cascade, complement and coagulation cascades, and fatty acid metabolism (**Figure 4A**), while the *NDRG2*-related signaling pathway is involved in fatty acid metabolism, oxidation by cytochrome p450, retinol metabolism, and eukaryotic translation elongation (**Figure 4B**).

### Correlation Between *NDRG1* or *NDRG2* Expression and Immune Infiltration

**Figure 5:**
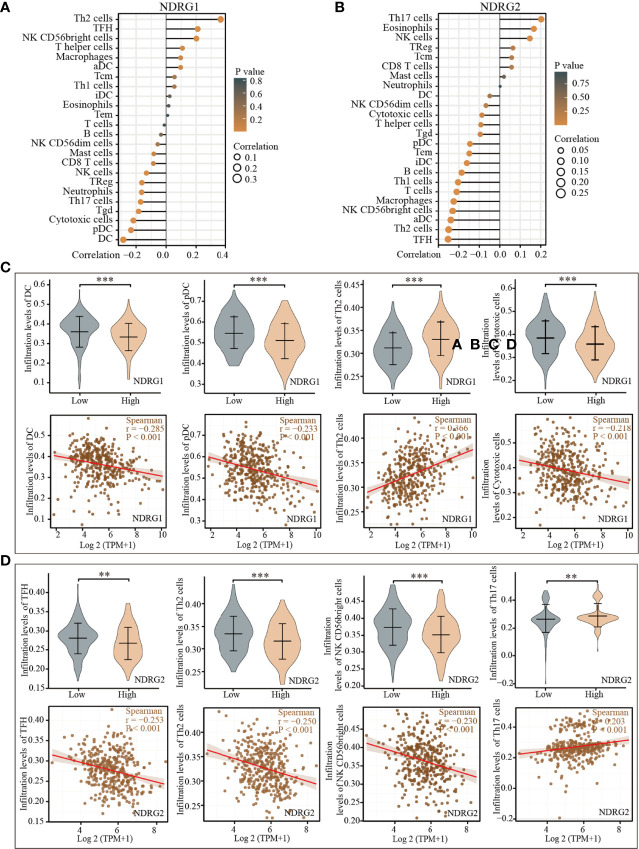
Correlation of the expression of *NDRG1 or NDRG2* with immune infiltration. **(A, B)** The forest plots showed the correlations of *NDRG1 or NDRG2* expression with immune infiltration in 24 types of immune cells. The size of the dots shows the absolute values of Spearman’s correlation coefficient. **(C, D)** Violin plots and scatter plots showing the difference and relevance of representative immune cells infiltration level. **P < 0.01; ***P < 0.001.

The tissue microenvironment is vital for tumor cells. Therefore, we first assessed the infiltration of 24 types of immune cells in LIHC using the ssGSEA method from the R package “GSVA”, and subsequently evaluated the relationship between *NDRG1* or *NDRG2* mRNA expression and immune cell infiltration by Spearman’s analysis. As shown in **Figure 5**, T helper cell 2 (Th2) (r = 0.366, *P <*0.001), follicular helper T cells (TFH) (r = 0.211, *P <*0.001), and NK CD56 bright cells (r = 0.202, *P <*0.001) showed a positive correlation with *NDRG1* expression, while dendritic cells (DC) (r = −0.285, *P <*0.001), cytotoxic cells (r = −0.218, *P <*0.001), and pre-dendritic cells (pDC) (r = −0.233, *P <*0.001) showed a negative correlation with *NDRG1* expression (**Figures 5A, C**). The expression of *NDRG2* was negatively correlated with TFH (r = −0.261, *P <*0.001), Th2 cells (r = −0.250, *P <*0.001), NK CD56 bright cells (r = −0.240, *P <*0.001), and positively correlated with T helper cell 17 (Th17) (r = 0.206, *P <*0.001) and NK cells (r = 0.147, *P <*0.004) (**Figures 5B, D**).

### Correlation Between *NDRG1* or *NDRG2* Expression and Clinicopathological Characteristics

The clinicopathological characteristics of LIHC patients with differential *NDRG1* and *NDRG2* expression are listed in **Table 1**. There was a significant difference in the distribution of BMI (*P* = 0.018), T stages (*P* = 0.031), pathologic stages (*P* = 0.016), residual tumor (*P* = 0.008), and histological grade (*P* = 0.027) between the high- and low-*NDRG1* groups. We also further evaluated the correlation between *NDRG1* or *NDRG2* expression and clinicopathological characteristics by logistic regression analysis (**Table 2**). As a result, we found markedly positive correlations of *NDRG1* expression with clinical T stage (*P* = 0.004), histological grade (*P* = 0.005), and AFP concentration (*P <*0.001).

**Table 1:** Demographics and tumor characteristics based on the expression of *NDRG1* and *NDRG2*.

Characteristic	levels	*NDRG1*		*P-*values	*NDRG2*		*P*-values
		Low(n = 185)	High(n = 186)		Low (n = 185)	High (n = 186)	
**Gender**				0.530			0.083
	Female	57 (15.4%)	64 (17.3%)		52 (14%)	69 (18.6%)	
	Male	128 (34.5%)	122 (32.9%)		133 (35.8%)	117 (31.5%)	
**Age**				0.076			0.469
	≤60	79 (21.4%)	98 (26.5%)		92 (24.9%)	85 (23%)	
	>60	105 (28.4%)	88 (23.8%)		92 (24.9%)	101 (27.3%)	
**BMI**				**0.018**			0.882
	≤25	79 (23.6%)	98 (29.3%)		91 (27.2%)	86 (25.7%)	
	>25	92 (27.5%)	66 (19.7%)		79 (23.6%)	79 (23.6%)	
**T stage**				**0.031**			0.301
	T1	97 (26.4%)	84 (22.8%)		82 (22.3%)	99 (26.9%)	
	T2	51 (13.9%)	43 (11.7%)		53 (14.4%)	41 (11.1%)	
	T3	28 (7.6%)	52 (14.1%)		43 (11.7%)	37 (10.1%)	
	T4	6 (1.6%)	7 (1.9%)		6 (1.6%)	7 (1.9%)	
**N stage**				0.622			0.350
	N0	126 (49.2%)	126 (49.2%)		133 (52%)	119 (46.5%)	
	N1	1 (0.4%)	3 (1.2%)		1 (0.4%)	3 (1.2%)	
**M stage**				1.000			0.624
	M0	130 (48.1%)	136 (50.4%)		138 (51.1%)	128 (47.4%)	
	M1	2 (0.7%)	2 (0.7%)		3 (1.1%)	1 (0.4%)	
**Pathologic stage**				**0.016**			0.142
	Stage I	90 (25.9%)	81 (23.3%)		79 (22.8%)	92 (26.5%)	
	Stage II	49 (14.1%)	37 (10.7%)		51 (14.7%)	35 (10.1%)	
	Stage III	30 (8.6%)	55 (15.9%)		43 (12.4%)	42 (12.1%)	
	Stage IV	3 (0.9%)	2 (0.6%)		4 (1.2%)	1 (0.3%)	
**Residual tumor**				**0.008**			0.542
	R0	167 (48.8%)	157 (45.9%)		161 (47.1%)	163 (47.7%)	
	R1	3 (0.9%)	14 (4.1%)		7 (2%)	10 (2.9%)	
	R2	0 (0%)	1 (0.3%)		1 (0.3%)	0 (0%)	
**Histologic grade**				**0.027**			**0.005**
	G1	31 (8.5%)	24 (6.6%)		19 (5.2%)	36 (9.8%)	
	G2	98 (26.8%)	79 (21.6%)		83 (22.7%)	94 (25.7%)	
	G3	51 (13.9%)	71 (19.4%)		74 (20.2%)	48 (13.1%)	
	G4	3 (0.8%)	9 (2.5%)		8 (2.2%)	4 (1.1%)	

Bold indicates significant differences.

**Table 2:** Correlation between *NDRG1* or *NDRG2* expression and clinicopathological characteristics in LIHC patients by logistic regression analysis.

Characteristics	Total(N)	*NDRG1*		*NDRG2*	
		Odds Ratio (OR)	*P*-values	Odds Ratio (OR)	*P*-values
Gender (Male vs. Female)	371	0.849 (0.549–1.311)	0.460	0.663 (0.427–1.025)	0.065
Age (>60 vs. ≤60)	370	0.676 (0.448–1.017)	0.061	1.188 (0.790–1.789)	0.408
BMI (>25 vs. ≤25)	335	0.578 (0.374–0.890)	**0.013**	1.058 (0.689–1.626)	0.796
T stage (T3 & T4 vs. T1 & T2)	368	2.022 (1.253–3.306)	**0.004**	0.866 (0.540–1.386)	0.549
N stage (N1 vs. N0)	256	3.000 (0.378–61.094)	0.344	3.353 (0.423–68.283)	0.298
M stage (M1 vs. M0)	270	0.956 (0.113–8.062)	0.964	0.359 (0.018–2.847)	0.378
Histologic grade (G3 & G4 vs. G1 & G2)	366	1.855 (1.208–2.867)	**0.005**	0.498 (0.321–0.765)	**0.002**
AFP (ng/ml) (>400 vs. ≤400)	278	4.090 (2.259–7.680)	**<0.001**	0.709 (0.402–1.237)	0.228
Vascular invasion (Yes vs. No)	315	1.409 (0.885–2.250)	0.149	0.660 (0.413–1.052)	0.082
Fibrosis ishak score (3/4&5/6 vs. 0&1/2)	212	1.131 (0.655–1.958)	0.659	1.253 (0.729–2.159)	0.416
Adjacent tissue inflammation (Mild & Severe vs. None)	234	1.584 (0.940–2.685)	0.085	0.499 (0.295–0.839)	**0.009**

Bold indicates significant differences.

### Diagnosis and Prognosis Values of *NDRG1* or *NDRG2* for LIHC

**Figure 6:**
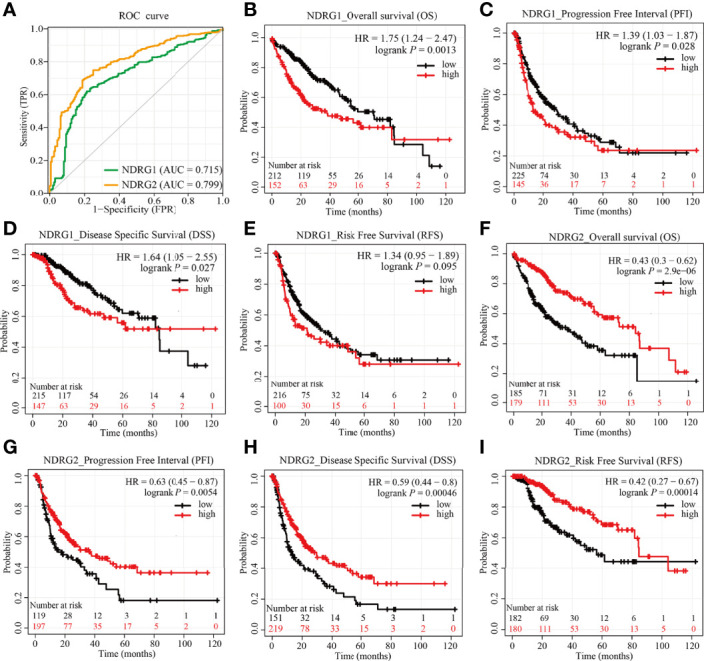
Predictive values of *NDRG1* or *NDRG2* for diagnosis and prognosis in LIHC. **(A)** AUROC analysis evaluating the diagnosis performance of *NDRG1* or *NDRG2* for LIHC between tumor and normal tissue. **(B–E)** Kaplan–Meier (KM) survival curves comparing *NDRG1*-high and -low patients with hepatocellular carcinoma. **(F–I)** KM survival curves of OS, PFI, DSS, and RFS between high and low expression groups of *NDRG2* in LIHC.

As shown in **Figure 6A**, the AUROC of *NDRG1* and *NDRG2* is 0.715 and 0.799, respectively. The results of AUROC indicated that the expression of *NDRG1* or *NDRG2* had high sensitivity and specificity for LIHC diagnosis. Next, Kaplan–Meier (K–M) analysis was performed to verify the prediction of *NDRG1* or *NDRG2* on clinical outcomes. As shown in **Figures 6B–E**, overall survival [OS, hazard ratio (HR): 1.75, *P* = 0.0013], disease-specific survival (DSS, HR: 1.64, *P* = 0.027), progression-free interval (PFI, HR: 1.39, *P* = 0.028), and risk-free survival (RFS, HR: 1.34, *P* = 0.095) for LIHC patients with high-*NDRG1* expression were statistically worse than those patients with the low-*NDRG1* expression. In contrast, OS (HR: 0.43, *P* = 2.9e−06), DSS (HR: 0.59, *P* = 0.00046), PFI (HR: 0.63, *P* = 0.0054), and RFS (HR: 0.42, *P* = 0.00014) for high-*NDRG2* expression groups were all statistically better than those of the low-*NDRG2* expression group (**Figures 6F–I**).

**Figure 7:**
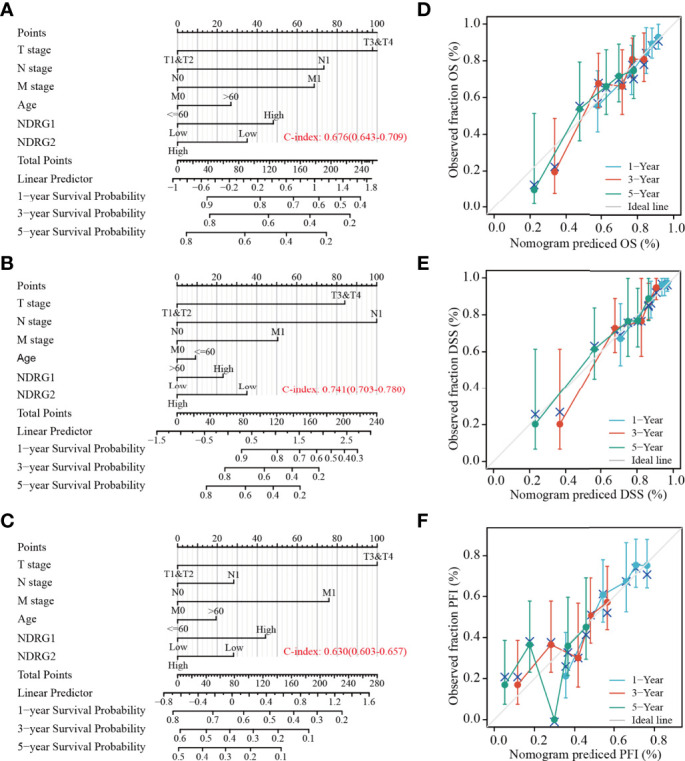
Construction and calibration of nomograms based on *NDRG1 or NDRG2* expression. **(A–C)** Risk scoring models for 1-, 3-, and 5-year overall survival (OS), disease-specific survival (DSS), and progression-free interval (PFI). **(D–F)** Calibration diagrams validating the efficiency of nomograms for OS, DSS, and PFI.

Subsequently, we constructed a prognostic nomogram using multivariate Cox regression analysis and validated the efficiency of the nomogram by drawing a calibration curve. As shown in **Figures 7A–C**, tumor TNM stage and age, as well as the expression level of *NDRG1* or *NDRG2*, were included in the nomogram to predict OS (C-index: 0.676, **Figure 7A**), DSS (C-index: 0.741, **Figure 7B**) and PFI (C-index: 0.630, **Figure 7C**). The calibration curves showed the desired predictive performance of these nomograms of 1-, 3-, and 5-year clinical outcomes (**Figures 7D–F**).

### Predictive Values of *NDRG1* or *NDRG2* For Clinicopathological Subgroups of LIHC

The LIHC patients were further divided into different clinicopathological subgroups according to sex, age, clinical TNM stage, and vascular invasion. Next, we also performed Cox regression analyses on each subgroup to assess the prognostic performance of *NDRG1* (**Table 3**) or *NDRG2* (**Table 4**) expression on OS, DSS, and PFI. As shown in **Table 3**, *NDRG1* was a significant risk factor for OS in male patients (HR = 1.97, *P* = 0.003), patients aged above 60 (HR = 1.78, *P* = 0.014), patients at N0 stage (HR = 1.55, *P* = 0.048), patients at M0 stage (HR = 1.60, *P* = 0.034), patients with vascular invasion (HR = 2.19, *P* = 0.028), and patients with tumor (HR = 1.58, *P* = 0.048). *NDRG1* was also a significant risk factor for DSS in patients with tumors (HR = 1.58, *P* = 0.048), and for PFI in females (HR = 2.55, *P* = 0.008), patients at T stage I–II (HR = 1.48, *P* = 0.034), and patients at N1 stage (HR = 1.44, *P* = 0.044).

**Table 3:** Prognostic performance of *NDRG1* on clinical prognosis in various liver cancer patient subgroups by Cox regression analysis.

Characteristics	N (%)	HR for overall survival (95% CI)	*P*	HR for disease-specific survival (95% CI)	*P*	HR for progression-free interval (95% CI)	*P*
**Sex**							
Female	121 (32.6%)	1.18 (0.68–2.05)	0.557	1.44 (0.70–2.93)	0.319	2.05 (1.21–3.47)	**0.008**
Male	250 (67.4%)	1.97 (1.25–3.11)	**0.003**	1.62 (0.91–2.86)	0.100	1.15 (0.80–1.65)	0.442
**Age**							
≤60	177 (47.8%)	1.3 (0.76–2.20)	0.339	1.19 (0.64–2.21)	0.585	1.27 (0.84–1.92)	0.266
>60	193 (52.2%)	1.78 (1.12–2.84)	**0.014**	1.8 (0.95–3.41)	0.072	1.49 (0.98–2.25)	0.060
**Clinical T stage**							
Stage I–II	275 (74.7%)	1.4 (0.89–2.21)	0.146	1.41 (0.76–2.61)	0.278	1.48 (1.03–2.13)	**0.034**
Stage III–IV	93 (25.3%)	1.65 (0.96–2.86)	0.071	1.32 (0.69–2.53)	0.397	1.29 (0.77–2.16)	0.336
**Clinical N stage**							
N0	252 (98.4%)	1.55 (1.00–2.40)	**0.048**	1.58 (0.90–2.77)	0.113	1.44 (1.01–2.06)	**0.044**
N1	4 (1.6%)	N.A.		N.A.		N.A.	
**Clinical M stage**							
M0	266 (98.5%)	1.6 (1.04–2.47)	**0.034**	1.42 (0.82–2.49)	0.213	1.38 (0.97–1.96)	0.073
M1	4 (1.5%)	N.A.		N.A.		N.A.	
**Vascular invasion**							
No	206 (65.4%)	1.21 (0.73–2.01)	0.465	1.11 (0.55–2.23)	0.766	1.48 (0.95–2.32)	0.082
Yes	109 (34.6%)	2.19 (1.09–4.41)	**0.028**	1.31 (0.50–3.40)	0.580	1.26 (0.75–2.12)	0.382
**Tumor status**							
Tumor free	201 (57.1%)	1.7 (0.92–3.15)	0.088	1.15 (0.87–1.51)	0.333	1.76 (0.82–3.76)	0.144
With tumor	151 (42.9%)	1.58 (1.00–2.49)	**0.048**	1.58 (1.00–2.49)	**0.048**	1.31 (0.94–1.82)	0.108

HR, hazard ratio; CI, confidence interval; N.A, Not Available. Bold indicates significant differences.

**Table 4:** Prognostic performance of *NDRG2* on clinical prognosis in various liver cancer patient subgroups by Cox regression analysis.

Characteristics	N (%)	HR for overall survival (95% CI)	*P-*values	HR for disease-specific survival (95% CI)	*P-*values	HR for progression-free interval (95% CI)	*P-*values
**Sex**							
Female	121 (32.6%)	0.87 (0.50–1.51)	0.611	1.10 (0.53–2.28)	0.796	0.99 (0.59–1.64)	0.959
Male	250 (67.4%)	0.62 (0.39–0.96)	**0.034**	0.53 (0.29–0.94)	**0.031**	0.79 (0.55–1.14)	0.209
**Age**							
≤60	177 (47.8%)	0.45 (0.26–0.78)	**0.004**	0.46 (0.24–0.88)	**0.019**	0.67 (0.44–1.01)	0.058
>60	193 (52.2%)	0.94 (0.59–1.47)	0.776	0.94 (0.50–1.76)	0.842	0.99 (0.650–1.49)	0.955
**Clinical T stage**							
Stage I–II	275 (74.7%)	0.68 (0.43–1.07)	0.095	0.73 (0.40–1.35)	0.320	0.88 (0.61–1.26)	0.493
Stage III–IV	93 (25.3%)	0.61 (0.35–1.06)	0.078	0.52 (0.27–1.01)	0.054	0.64 (0.38–1.08)	0.092
**Clinical N stage**							
N0	252 (98.4%)	0.65 (0.42–1.01)	0.054	0.53 (0.30–0.94)	**0.029**	0.81 (0.56–1.15)	0.236
N1	4 (1.6%)	N.A.		N.A.		N.A.	
**Clinical M stage**							
M0	266 (98.5%)	0.60 (0.39–0.92)	**0.020**	0.56 (0.32–0.98)	**0.043**	0.82 (0.58–1.17)	0.275
M1	4 (1.5%)	N.A.		N.A.		N.A.	
**Vascular invasion**							
No	206 (65.4%)	0.67 (0.40–1.13)	0.134	0.76 (0.37–1.54)	0.443	0.88 (0.56–1.36)	0.556
Yes	109 (34.6%)	0.96 (0.49–1.89)	0.911	0.85 (0.33–2.21)	0.745	1.02 (0.61–1.73)	0.931
**Tumor status**							
Tumor free	201 (57.1%)	0.71 (0.39–1.30)	0.261	0.79 (0.60–1.05)	0.101	1.09 (0.51–2.31)	0.825
With tumor	151 (42.9%)	0.72 (0.46–1.14)	0.161	0.72 (0.46–1.14)	0.161	0.93 (0.67–1.28)	0.108

HR, hazard ratio; CI, confidence interval; N.A, Not Available. Bold indicates significant differences.

As shown in **Table 4**, *NDRG2* was a significant favorable factor for OS in male patients (HR = 0.62, *P* = 0.034), patients aged below 60 (HR = 0.45, *P* = 0.004), and patients at M0 stage (HR = 0.60, *P* = 0.020). Similar observations occurred for DSS in male patients (HR = 0.53, *P* = 0.031), patients aged below 60 (HR = 0.46, *P* = 0.019), patients at the N0 stage (HR = 0.53, *P* = 0.029), and patients at the M0 stage (HR = 0.56, *P* = 0.043). All the results demonstrated significantly better clinical outcomes in the low-*NDRG1* or high-*NDRG2* expression groups.

## Discussion

To date, clinical outcomes of LIHC are far from satisfactory owing to the lack of efficient indicators and effective treatment (26). Therefore, it is crucial to find potential biomarkers for predicting clinical prognosis and guiding individualized treatment. Many cancers have been reported to have the N-myc downstream-regulated gene (*NDRG1–4*) family. To our knowledge, the expression levels of *NDRG* family members and their potential prognostic values in LIHC have not been fully explored. Hence, we focused on the expression and prognostic values of *NDRG1* and *NDRG2* in LIHC by analyzing datasets from the TCGA and GEO.

Here, the data analysis from UCSC XENA indicated that the *NDRG1* expression had a significant increase in LIHC tumor tissues compared to normal tissues (*P <*0.001). The same result can be found in the GEO database (GSE14520, GSE25097, and GSE36376), the HPA database, and our experiment validation (qPCR). N*DRG1* is a member of the *NDRG* superfamily and has been found to be involved in embryonic development (7), cellular vesicle transportation (27), and membrane protein circulation (28). *NDRG1* is strongly upregulated under hypoxic conditions, and this condition is prevalent in solid tumors (29). Previous studies suggested that *NDRG1* could inhibit proliferation and induce apoptosis of cancer cells by regulating Bcl-2 and Ca^2+^-associated proteins (30, 31) and epithelial–mesenchymal transition (EMT) (32). Another study indicated that the encoded protein of *NDRG1* is necessary for p53-mediated caspase activation and apoptosis (33). However, *NDRG1* protein is expressed at low levels in normal tissues while *NDRG1* mRNA is ubiquitously over-expressed in various human cancers. In other words, the regulation mechanism involved in *NDRG1* is somewhat complex, governed by hypoxia-inducible factor 1 alpha (HIF-1α)- and p53-dependent pathways (29), which makes the *NDRG1* gene potentially an important biomarker for tumor progression.

Furthermore, Kaplan–Meier analysis showed that high *NDRG1* expression correlates significantly with poor clinical outcomes in LIHC patients, which was consistent with previous research (34). Our results suggest that *NDRG1* is an independent risk factor for LIHC and could act as an oncogene to accelerate LIHC progression. However, previous studies revealed that the expression of *NDRG1* decreased in esophageal (16), colorectal (35), and breast cancers (10), and was correlated with poor clinical outcomes. This contradictory result may be tumor type-specific, further emphasizing that *NDRG1* is involved in complex biological processes. In contrast to *NDRG1*, *NDRG2* expression was reduced significantly in LIHC, and the downregulation of *NDRG2* was associated with poor prognosis, which was consistent with previous studies (9, 14). Past research has also shown that the *NDRG2* expression has a significant decrease in gastric (36), pancreatic (14), and breast cancers (15). Our results showed that *NDRG2* might act as an antioncogene to suppress the LIHC progression.

Different types of mutations can greatly increase the risk of developing certain cancers. Interestingly, we found that the *NDRG1* gene has a mutation frequency of up to 14% in LIHC patients, while the other *NDRG* family members have a mutation frequency of less than 2%. DNA methylation is a common epigenetic phenotype that exists in almost all types of human cancers (37), and its occurrence in the promoter region often results in gene silencing (38). In this study, the methylation of *NDRG1* and *NDRG2* was related to the clinical prognosis of LIHC patients, and patients with hypomethylated *NDRG1* or hypermethylated *NDRG2* had worse OS. Of note, their mRNA expression was consistent with DNA methylation levels for clinical prognosis. Therefore, the promoter methylation status and mRNA levels of *NDRG1* and *NDRG2* can be used as independent predictors of LIHC patients.

The tumor microenvironment (TME), composed of diverse cell populations in a complex matrix, plays a crucial role in the occurrence and progression of tumors. Tumor-associated fibroblasts in TME contribute to the progression of LIHC by secreting various growth factors and cytokines (39). Tumor-associated immune cells in TME could be divided into tumor-antagonizing and tumor-promoting immune cells (40). In this study, we observed that *NDRG1* and *NDRG2* expression were negatively associated with most of the immune infiltration cells in LIHC, namely, DCs, macrophages, and neutrophils. As is known to all, DCs are the most effective antigen-presenting cells, which can activate CD8^+^ T cells and then initiate anti-tumor immunity (41). In the following immune response, neutrophils and macrophages work together against tumors (42). Therefore, the upregulation of *NDRG1* or downregulation of *NDRG2* seemed to suppress tumor immunity, assist cancer cells to escape from immune elimination, and finally promote tumorigenesis. Meanwhile, *NDRG1* mRNA level was positively associated with Th2 cell infiltration level, and *NDRG2* expression was positively correlated with Th17 and NK cell infiltration level. These results indicate that *NDRG1* and *NDRG2* may play vital roles in tumor infiltration immunity. Here, we observed the correlations between immune cell infiltration and the expression of *NDRG1* and *NDRG2*. However, there remains a gap to be filled between *NDRG1* and *NDRG2* in LIHC cancer and various types of immune cells.

The clinical significance of *NDRG1* and *NDRG2* is another concern. Our AUROC provided strong evidence that *NDRG1* or *NDRG2* could be biomarkers for LIHC diagnosis and prognosis. Further Cox regression analysis and nomograms further demonstrated that *NDRG1* or *NDRG2* had promising performance for evaluating clinical outcomes. Patients with higher *NDRG1* or lower *NDRG2* levels had worse OS, PFI, and DSS. In particular, the prognostic value of *NDRG1* or *NDRG2* was better when considering gender, age, and clinical TNM stages (**Figure 7**). As a result, *NDRG1* or *NDRG2* could be a promising biomarker for poor prognosis prediction. Our findings are consistent with previous reports that *NDRG1* plays a role in promoting tumorigenesis in liver, kidney, and esophageal cancers (43). In contrast, evidence has elucidated that *NDRG1* is associated with anti-oncogenic and anti-metastatic effects in breast, prostate, colorectum, and pancreatic cancers (44). The inconsistent results might be that *NDRG1* is potentially involved in many biological processes and the bi-directional crosstalk and may not play one single role. The pleiotropy of NDRG1 may reflect the heterogeneity of signal transduction in different tumor cell-types. Nowadays, the multi-omics approach (45) and the emerging single-cell studies (46) provide a new perspective on identifying biomarkers for clinical diagnosis and tumor typing.

In short, the mRNA expression and DNA methylation of NDRG superfamily members (*NDRG1* and *NDRG2*) show the potential for LIHC diagnosis and prognosis *via* integrative analysis from multiple cohorts. Considering the multiple mechanisms of the *NDRG* family, more experiments and a larger sample size will be needed to demonstrate and validate our bioinformatics results for future clinical application.

## Data Availability Statement

The original contributions presented in the study are included in the article/supplementary material. Further inquiries can be directed to the corresponding author.

## Ethics Statement

The studies involving human participants were reviewed and approved by The hospital ethics committee, Zhongnan Hospital of Wuhan University. The patients/participants provided their written informed consent to participate in this study. Written informed consent was obtained from the individual(s) for the publication of any potentially identifiable images or data included in this article.

## Author Contributions

S-ML and SX conceived and designed the experiments. SX, YZho, YY, and RG analyzed data and drafted the original manuscript. YZha and RG performed the experiments. S-ML, CL and QL critically revised the manuscript. All authors listed have made a substantial, direct, and intellectual contribution to the work and approved it for publication.

## Funding

This work was supported by the National Natural Science Foundation of China (81772276) and the Hubei Provincial Natural Science Fund for Creative Research Groups (2019CFA018).

## Conflict of Interest

The authors declare that the research was conducted in the absence of any commercial or financial relationships that could be construed as a potential conflict of interest.

## Publisher’s Note

All claims expressed in this article are solely those of the authors and do not necessarily represent those of their affiliated organizations, or those of the publisher, the editors and the reviewers. Any product that may be evaluated in this article, or claim that may be made by its manufacturer, is not guaranteed or endorsed by the publisher.
